# Effect of latent cytomegalovirus infection on the antibody response to influenza vaccination: a systematic review and meta-analysis

**DOI:** 10.1007/s00430-019-00602-z

**Published:** 2019-04-04

**Authors:** S. P. H. van den Berg, K. Warmink, J. A. M. Borghans, M. J. Knol, D. van Baarle

**Affiliations:** 1 Centre for Infectious Disease Control, Immunology of Infectious Diseases and Vaccines, National Institute for Public Health and the Environment, Bilthoven, The Netherlands; 2 Laboratory of Translational Immunology, University Medical Center Utrecht, Utrecht, The Netherlands; 3 Centre for Infectious Disease Control, Epidemiology and Surveillance Unit, National Institute for Public Health and the Environment, Bilthoven, The Netherlands

**Keywords:** Cytomegalovirus, Influenza, Vaccine, Immunosenescence, Age, Antibody response

## Abstract

Latent infection with cytomegalovirus (CMV) is thought to accelerate aging of the immune system. With age, influenza vaccine responses are impaired. Although several studies investigated the effect of CMV infection on antibody responses to influenza vaccination, this led to contradicting conclusions. Therefore, we investigated the relation between CMV infection and the antibody response to influenza vaccination by performing a systematic review and meta-analysis. All studies on the antibody response to influenza vaccination in association with CMV infection were included (*n* = 17). The following outcome variables were extracted: (a) the geometric mean titer pre-/post-vaccination ratio (GMR) per CMV serostatus group, and in addition (b) the percentage of subjects with a response per CMV serostatus group and (c) the association between influenza- and CMV-specific antibody titers. The influenza-specific GMR revealed no clear evidence for an effect of CMV seropositivity on the influenza vaccine response in young or old individuals. Meta-analysis of the response rate to influenza vaccination showed a non-significant trend towards a negative effect of CMV seropositivity. However, funnel plot analysis suggests that this is a consequence of publication bias. A weak negative association between CMV antibody titers and influenza antibody titers was reported in several studies, but associations could not be analyzed systematically due to the variety of outcome variables. In conclusion, by systematically integrating the available studies, we show that there is no unequivocal evidence that latent CMV infection affects the influenza antibody response to vaccination. Further studies, including the level of CMV antibodies, are required to settle on the potential influence of latent CMV infection on the influenza vaccine response.

**Electronic supplementary material:**

The online version of this article (10.1007/s00430-019-00602-z) contains supplementary material, which is available to authorized users.

## Introduction

Age-related reduced function of the immune system, often referred to as “immunosenescence”, is suggested to be influenced by cytomegalovirus (CMV) infection [1]. Main features of CMV seropositivity include low percentages of naïve T-cells and reduced diversity in the T cell repertoire, which may impair the ability to respond to heterologous infection or vaccination [2] and result in lower B-cell functions by lack of T-cell help [3, 4]. CMV seropositivity has also been identified as a factor of the Immune Risk Profile (IRP) for mortality in the Swedish longevity studies [5].

CMV prevalence increases with age in the general population from 30% in children to above 90% at the age of 80 and older [6, 7]. Primary CMV infection and reactivation from latency can cause significant problems when the immune system is compromised or immature, but is usually asymptomatic in healthy individuals [8]. However, CMV frequently reactivates during life [9, 10] and can lead to detectable CMV DNA levels, mainly in the elderly [8, 11]. Control of CMV requires continuous immune surveillance and leads to large numbers of CMV-specific T cells, up to 10–30% of CD8+ T cells in the periphery. The lifelong need to control CMV is by many thought to take its toll and to hamper immune responses to heterologous infections or vaccination [3]. Indeed, in several mouse models the immune responses to heterologous infections was shown to be negatively affected by CMV [12–14]. However, other studies suggested a positive effect of CMV on the response to heterologous infections [12, 15].

In humans, the potential effect of CMV-infection on a heterologous immune response is mainly studied in the context of influenza vaccination. Seasonal influenza vaccination is an effective means to prevent influenza infection [16–18]. However, effectiveness of influenza vaccination decreases with age, leaving older adults exposed to an increased risk of influenza infection [1]. In older adults, influenza infection more often leads to disease-related hospitalization, complications and mortality [17, 19–21]. Influenza vaccines are primarily focused on eliciting a strain-specific antibody response. Antibodies are important as they give rise to so-called sterilizing immunity; the immune status where the host immune response effectively blocks virus infection. The most widely used method to measure strain-specific influenza titers is the hemagglutination-inhibition (HI) assay, which reflects the ability of specific antibodies to bind influenza virus and inhibit viral agglutination of red blood cells [22, 23]. European medicine agency guidelines describe the analysis and presentation of influenza antibody data for development of influenza vaccines [24], stating as a minimum requirement that geometric mean titers (GMTs) (with 95% confidence intervals) and pre-/post-vaccination ratios (GMR), and response rates should be reported.

Clarification of the effect of CMV on influenza vaccine responses is of high importance. The current suboptimal immune response to influenza vaccination in elderly will become an increasingly large problem. By 2050, the population of older persons (defined by the United Nations as those aged 60 years and above) is expected to double in size compared to 2015. With an increasing life expectancy, the group of elderly at high-risk for influenza complications will increase quickly and contribute to the rising challenges of public health. As latent CMV infection is highly frequent in the population, it is critical to elucidate whether CMV infection influences influenza vaccination responses, to be able to optimize vaccine strategies in the population.

Several studies investigated the effect of CMV infection on immune responses induced by influenza vaccination. The first study by Trzonkowski et al. reported a negative association between CMV-infection and the response to influenza vaccination [25]. Some studies confirmed this result [26], but others did not find an effect of CMV infection on the influenza vaccine response [27]. In contrast, Furman et al. reported a positive effect of CMV infection on the immune response to influenza vaccination in adults [28]. To date, no consensus of the effect of latent CMV infection on the antibody response to influenza vaccination has been reached [4, 29, 30].

Here, we systematically reviewed studies on the effect of CMV infection on the antibody response to influenza vaccination in healthy individuals. The process of systematic reviewing the available evidence in literature was reported in line with the PRISMA criteria (Preferred Reporting Items for Systematic Reviews and Meta-Analyses) [31]. We extracted three standardized outcome variables of 17 studies, in line with European Medicine Agency (EMA) [24] and Food and Drug Administration (FDA) guidelines [32]. Summarizing all extracted data on GMR to influenza vaccination revealed no clear difference between CMV-seropositive and CMV-seronegative individuals. In a meta-analysis, a small (but non-significant) trend was observed that CMV-seropositive participants responded less often to influenza vaccination than CMV-seronegative individuals. We show that this effect is likely explained by publication bias. In addition, we summarized reports on a possible correlation between CMV antibody titers and influenza antibody titers, which showed weak negative correlations between the two. Together, these analyses provide no unequivocal evidence that latent CMV infection affects the influenza antibody response to vaccination.

## Materials and methods

### Search strategy and selection criteria

The database EMBASE was systematically searched for articles on CMV and influenza vaccination, combined with a search on CMV and immune response to include articles that covered the subject but did not explicitly mention influenza vaccination. The full search strategy was performed on 27-06-2017 and is provided in Supplementary Table 1. Two authors independently performed the selection process (SB and KW), in which all identified articles were first screened based on title and abstract and the remaining articles were reviewed in depth. Discrepancies regarding the inclusion or exclusion of an article between the authors were resolved by discussion. English articles that reported an immunological response to influenza vaccination and had included at least a CMV-seronegative group or a CMV-seropositive group were considered for inclusion.

No restrictions were placed on study design or publication date. Only human studies with in vivo influenza vaccination were included. No restriction was placed on the age of the study population. Studies on primary CMV infection, CMV disease or immunocompromised participants were not included, because the immune system is expected to operate differently in those cases.

### Data extraction and risk of bias assessment

Data collected from the studies included study design, study population, the type of influenza vaccine and the reported outcomes. These data were extracted via a data-extraction form, which was developed by KW based on the Cochrane Data Extraction and Assessment Template [33]. The form was pre-tested on several articles by KW and MK and refined accordingly. The final form can be found in Supplementary Table 2. If influenza vaccine response outcomes of several studies were reported in one article, the studies were assessed as separate studies.

The quality of each individual study was investigated by assessment of the risk of bias based on the Newcastle–Ottawa scale for cohort studies [34]. According to these guidelines, studies were awarded with “stars” for high quality choices in three categories: “selection of cohorts” (max 4*), “comparability of cohorts” (max 2*) and “assessment of outcome” (max 3*). Based on all the acquired information, a study could acquire a maximum of 9 stars and the overall quality of the study was rated as high (+) (≥ 8 stars), intermediate (+/−) (7 stars) or low (−) (≤ 6 stars).

### Data analysis: statistical and narrative synthesis

**Fig. 1:**
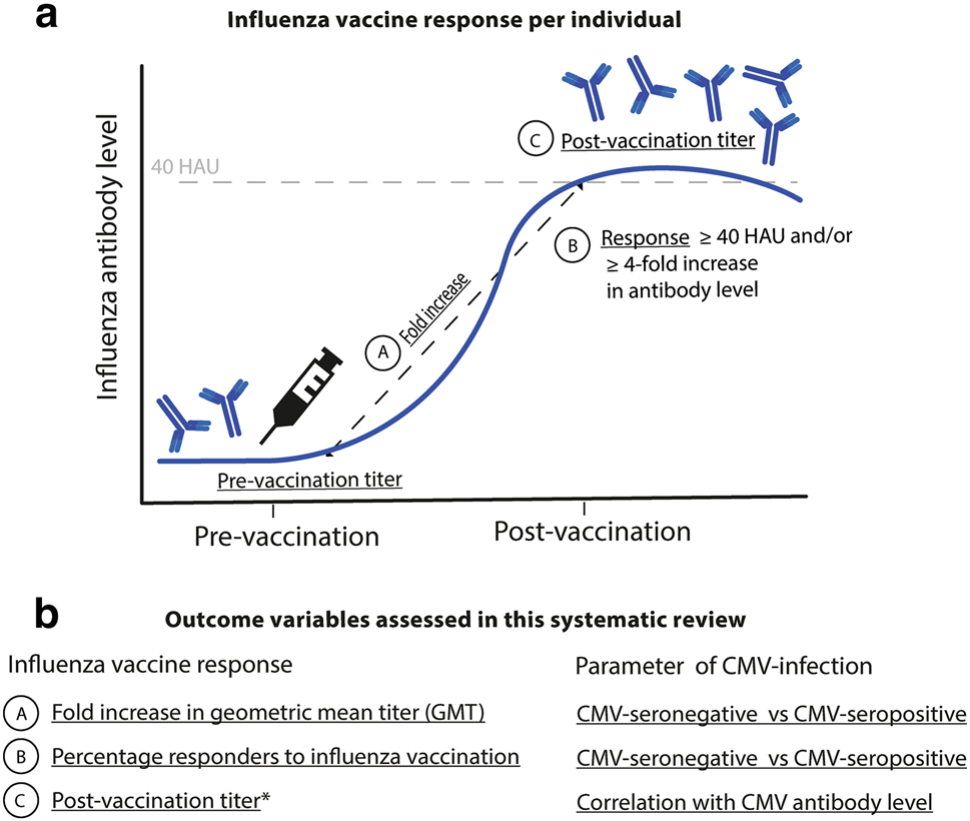
Investigated influenza antibody outcomes. The influenza antibody vaccine response is investigated in the context of CMV infection in this review in three ways, based on the variables as indicated by A, B and C. Outcomes are (a) the geometric mean titer pre-/post-vaccination ratio (GMR) per CMV serostatus group, (b) the percentage of subjects with a response per CMV serostatus group and (c) the association between the post-vaccination influenza antibody titers and CMV antibody titers. *1 study reporting correlations (outcome c) did not correlate the post-vaccination titer, but the fold increase. HAU, hemagglutination unit. 40 HAU = correlate of protection

As the outcome variables were heterogeneous, a combination of narrative and statistical approaches to data synthesis was applied. Three influenza antibody outcomes were systematically extracted from the studies (Fig. 1), in line with European Medicine Agency (EMA) guidelines for handling of influenza antibody data. Following the EMA guidelines, whenever possible, we separately extracted the outcome variables per influenza strain. Since age is the most important confounder, we also extracted the three outcome variables separately for young and old individuals. Findings were reported per outcome variable.

The principal outcome variable that we studied was the influenza-specific geometric mean titer ratio (GMR) pre-/post-vaccination (outcome a) with corresponding 95% confidence interval (CI) in CMV-seropositive and CMV-seronegative participants. Studies that reported this outcome or reported the data required to calculate the outcome were included in a figure per age subgroup. Per study the definition of young and old individuals differed, leading to two age groups in this analysis: young (< 65 years of age) and old individuals (> 60 years of age). Studies of which corresponding 95% CI could not be extracted or calculated reliably were summarized in a separate figure, also per age subgroup (young and old adults).

Secondly, the odds ratio (OR) with 95% CI for the association between a response to influenza vaccination and CMV serotatus was investigated (outcome b). The ORs were calculated using the numbers of responders and non-responders presented in the studies, where response to vaccination was defined as a ≥ fourfold increase, a post-vaccination titer ≥ 40 hemagglutinating units (HAU), or both. Meta-analysis was performed in R 3.3.3 using the ‘metafor’ package [35], to compare and pool the ORs of the studies. Random effects meta-analysis was performed in R 3.3.3 using the ‘metafor’ package [35]. The pooled OR and 95% CI using the DerSimonian–Laird method were calculated for the total group and for the predefined subgroups of young (< 60 years of age) and old (> 60 years of age) participants [36]. Heterogeneity among studies was assessed by the *χ*^2^-based *Q* test and *I*^2^ statistics. To analyze the influence of the quality of the studies, a sensitivity analysis was done by calculating the pooled OR for the highest-quality studies which had least potential for bias and confounding. The presence of possible publication bias was assessed using funnel plot regression [37]. The possible presence of any undetected studies and an effect estimate adjusted for publication bias were calculated using the trim-and-fill function in the ‘metafor’ package [38] in R 3.3.3.

Thirdly, the role of CMV antibody levels, instead of CMV serostatus, was investigated by extracting associations between influenza antibody titers and CMV antibody levels (outcome c). Correlations between CMV antibody levels and influenza antibody titers were tabulated, and outcomes of regression models incorporating CMV antibody levels and influenza antibody titers were narratively synthesized. No restriction for this outcome was placed on reporting post-vaccination titers or fold increase influenza titers to vaccination. Likewise, no restriction was placed on the performed statistics.

## Results

### Selection and quality assessment of retrieved articles

#### Study selection and characteristics

**Fig. 2:**
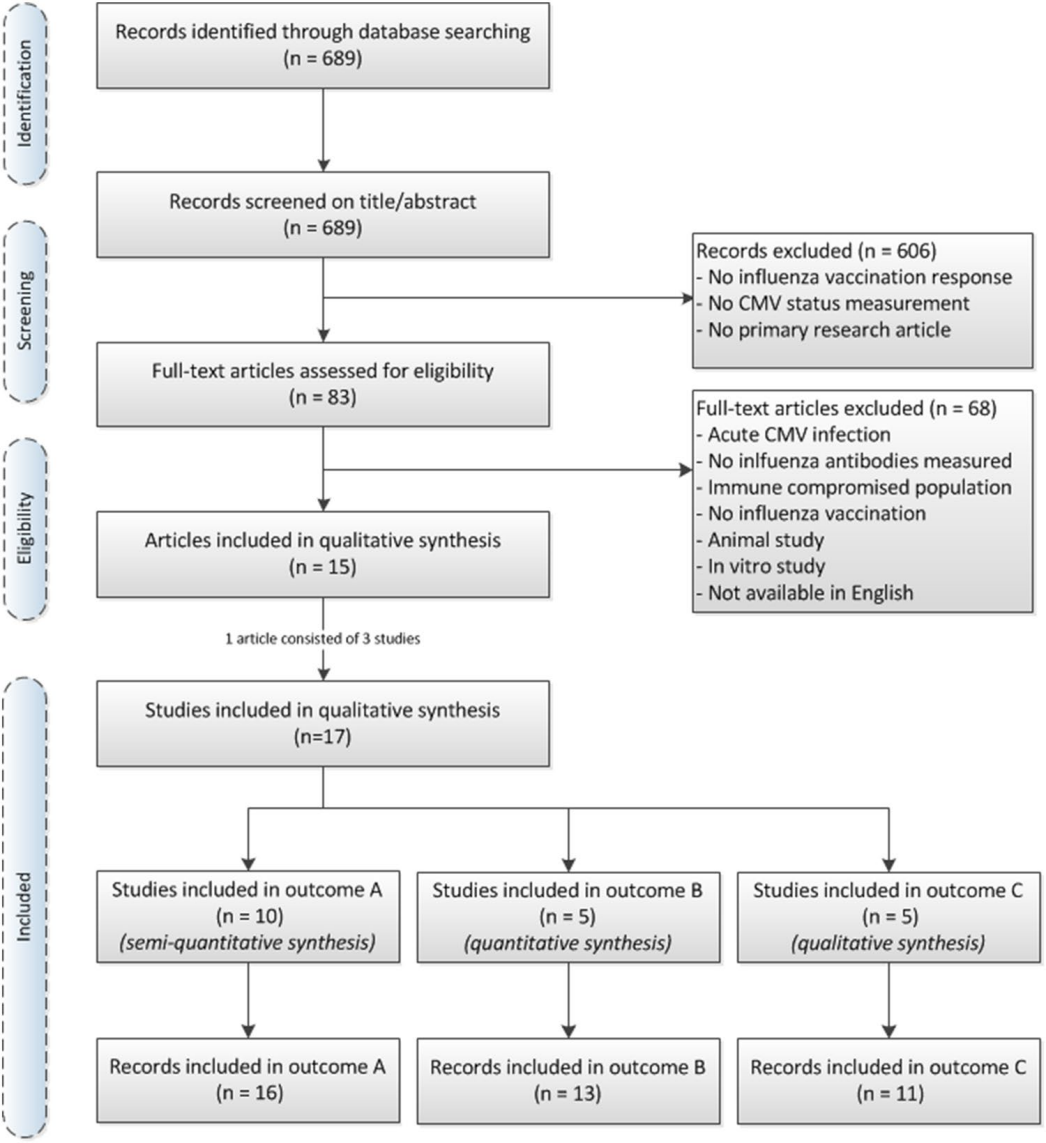
PRISMA flow diagram of identification and selection of studies

The comprehensive EMBASE search for articles on CMV and influenza vaccination retrieved 689 individual publications (Fig. 2). The first selection based on screening of title and abstract reduced this to 83 articles. Reasons for exclusion were for example no reporting of influenza vaccination response, no CMV serostatus measurement or no primary research article. The selected 83 articles were assessed in full text and 15 articles of these were included in this systematic review. Articles were excluded if the data came from acute CMV infection or CMV disease or from an immune compromised population, or when CMV infection was only studied in vitro or in an animal model. The agreement on the independently performed study selection between authors KW and SB was large with disagreement on only three studies. These differences could easily be resolved by discussion and judgement by a third person was not needed. References of the 15 included articles were checked for additional relevant articles, but none were found. One article (Furman et al.), that contained the outcomes of three different study populations, was assessed as three individual studies, which brought the total number of studies up to 17. Whenever possible, study records were stratified by influenza strains (H1N1, H3N2, Influenza B) and age groups (young and old), which led to multiple records per outcome variable for some studies (Supplementary figures 1–4).

Characteristics of the retrieved studies are summarized in Table 1. Most studies were cohort studies, but some were primarily set up as a vaccine trial with a subgroup analysis for CMV serostatus. For our research question, all studies could be considered as observational studies. The sample size differed between studies with a range from 37 to 731 participants, leading to a total of 2249 participants. The age groups differed between studies with a range of 19–97 years of age and in some studies both young and older adults were included [25, 26, 39–42]. The definition of older adults differed between studies. In this review, either > 60 years or > 65 years of age was used, as indicated per subgroup or outcome variable.

**Table 1:**
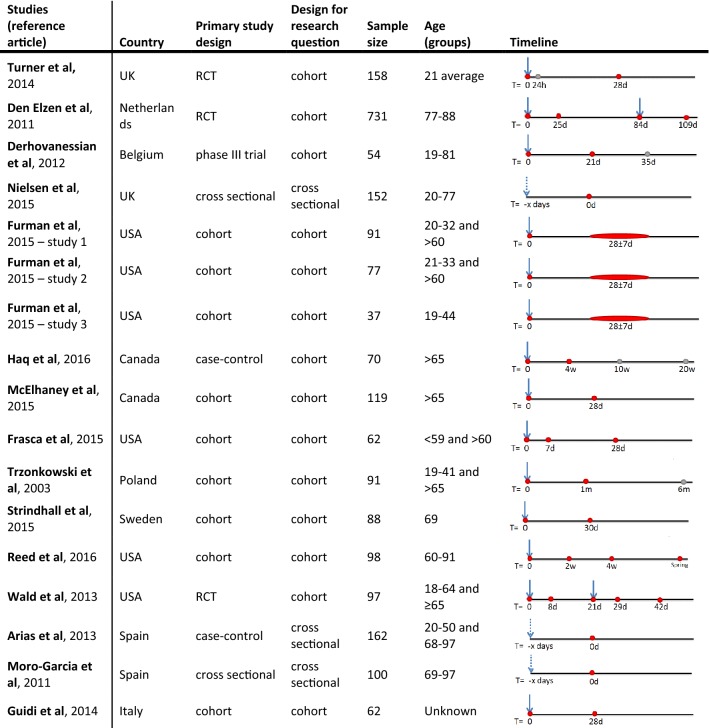
Characteristics of included studies. Timeline represents moment of vaccination (arrow) and blood withdrawn (red dot) on which influenza vaccine response is investigated or other information is gathered (gray dot). Studies of Moro-Garcia et al. and Arias et al. seem to use a population of the same elderly cohort

#### Quality assessment of studies

The risk of bias assessment led to an overall quality score per study. Twelve studies were rated as having a high quality, one an intermediate quality and four a low quality (Table 2). Most studies scored high on the selection process (1) with good representativeness of the exposed cohort and selection of the non-exposed cohort from the same population. Many different ways of reporting the influenza vaccine outcome were used and the quality of the reported outcome varied between the studies. All cohort studies measured the outcome of interest also before vaccination (pre-vaccination titers), which granted them an additional star compared to the three cross-sectional studies. In five studies, influenza titers were assessed using ELISA, which is generally regarded a less reliable method than the hemagglutination inhibition (HI) assay [43]. All studies had an adequate follow-up time, which was considered to be more than 2 weeks for humoral immunity outcomes [44, 45].

**Table 2:** Risk of bias and study quality of studies included for systematic assessment

Study	Selection (max 4*)	Comparability (max 2*)	Outcome (max 3*)	Overall quality
Turner et al. (2014)	****	**	***	+
Den Elzen et al. (2011)	****	**	***	+
Derhovanessian et al. (2012)	****	**	***	+
Nielsen et al. (2015)	***	–	*	−
Furman et al. (2015)—study 1	****	**	***	+
Furman et al. (2015)—study 2	****	**	***	+
Furman et al. (2015)—study 3	***	**	**	+/−
Haq et al. (2016)	****	**	***	+
McElhaney et al. (2015)	***	**	***	+
Frasca et al. (2015)	****	**	***	+
Trzonkowski et al. (2003)	****	**	**	+
Strindhall et al. (2015)	***	**	***	+
Reed et al. (2016)	****	**	**	+
Wald et al. (2013)	****	**	***	+
Arias et al. (2013)	***	**	*	−
Moro-Garcia et al. (2011)	***	*	*	−
Guidi et al. (2014)	***	–	***	−

Risk of bias was analyzed based on the Newcastle–Ottawa scale for cohort studies. According to these guidelines, studies were awarded with “stars” for high quality choices in three categories: “selection of cohorts” (max 4*), “comparability of cohorts” (max 2*) and “assessment of outcome” (max 3*). Based on all the acquired information, a study could acquire a maximum of 9 stars and the overall quality of the study was rated as high (+) (≥ 8 stars), intermediate (+/−) (7 stars) or low (−) (≤ 6 stars)

#### Non-systematic summary of conclusions reported in the different studies

**Fig. 3:**
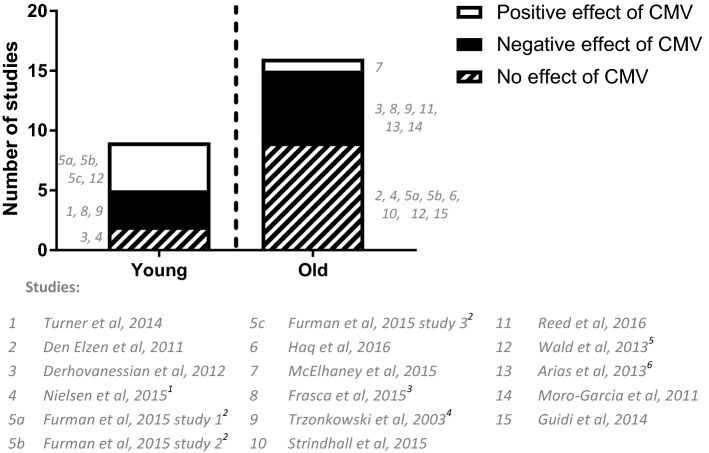
Summary of conclusions from studies on latent CMV infection on the influenza antibody response. Conclusion per study are shown for the effect of CMV-infection on the influenza antibody response, separated for young or old individuals. Per study the definition of young and old individuals differed, as indicated in Table 1. A flow diagram of records available per reported conclusion out of the 15 articles is presented in supplementary figure 1. Note that the article of Furman et al. contained the outcome of three study populations and was thus assessed as three individual studies. The study group in Nielsen et al. had an age range of 21–77 years, covering both young and old adults; therefore, the reported conclusion (no effect) was included in both young and old bar graphs. Statistics per study were performed by parametric tests on log-transformed influenza antibody data, unless indicated otherwise in the following notes. ^1^A Mann–Whitney test was performed on raw influenza antibody data (post-titer). ^2^Data were presented as a geometric mean of three different influenza strains titer. Also, we could not verify how the geometric mean of three influenza strains per individual was handled in the measurement of spread on group level. ^3^A Mann–Whitney test was performed on fold increase of influenza antibody data. ^4^Antibody data were analyzed with non-parametric test (Spearman correlation) on non-log-transformed antibody data (for both CMV and influenza antibodies). ^5^We could not verify what statistics were used, since it is stated in the paper that the GMT and 95% CI of day 21 post-vaccination are presented in Fig. 1 of the article, but the 95% CI showed equally distributed error bars on a linear scale. Also, it is stated that Mann–Whitney was used to compare GMTs, which is statistically not possible

First, we summarized the conclusions on a possible effect of CMV-infection on the antibody response to influenza vaccination reported in the different studies that we included (Supplementary Table 3 and Figure 3) for young (< 60 years of age) or old (> 60 years of age) adults. In young individuals, two studies reported no effect, three a negative effect and four a positive effect of CMV seropositivity. In old individuals, nine studies reported no effect, six a negative effect and one a positive effect of CMV seropositivity on the humoral influenza vaccination response. Overall, more studies investigated old individuals than young individuals and a positive effect of CMV seropositivity was mainly reported in young individuals. When reviewing the results based on strain type or influenza antibody outcome variable, no clear conclusion could be drawn (data not shown).

Assessment of the statistical methods performed by the studies showed that the majority of the studies used appropriate statistics, but different methods were applied for the statistical testing of influenza antibody data (legend Fig. 3). Normalization of HI data by log transformation and parametrical testing to compare the geometric titer is preferred [24, 46]. However, some studies performed non-parametric testing on raw antibody data to compare the median, which can be different from the geometric mean, especially in cases where the raw data are not natural log-distributed. In addition, influenza antibody outcome variables differed greatly between studies (Table 3). This hampers direct comparison of the results of the different studies in the literature and a systematic comparison of the studies is necessary.

**Table 3:** Data extraction and conversion of the three outcomes investigated in this review

Influenza antibody response	Turner et al.	Den Elzen et al.	Derhovanessian et al.	Nielsen et al.	Furman et al. study 1	Furman et al. study 2	Furman et al. study 3	Haq et al.	McElhaney et al.	Frasca et al.	Trzonkowski et al.	Strindhall et al.	Reed et al.	Wald et al.	Arias et al.	Moro-Garcia et al.	Guidi et al.
GMR (outcome a)	✔^1,2,3^	✔^8^			✔^11,12^	✔^11,12^	✔^11,12^	✔^13^	✔^14^	✔^15,16^		✔		✔^21^	✔^23,24^		✔^27^
Response rate (outcome b)	✔^3,4^	✔	✔							✔^15,16^		✔^19^		✔^22^			✔
Correlation (outcome c)	✔^3,5,6^										✔^17^				✔^25^	✔^22,23,26^	
Another outcome				✔^10^							✔^18^						
Model		✔^9^											✔^20^				

Per study on the effect of CMV-infection on the influenza antibody response to influenza vaccination is indicated what outcome is reported. Numbers refer to footnotes presented in the textbox. ✔Outome is reported. GMR: the geometric mean titer pre-/post-vaccination ratio (GMR) per CMV serostatus group. Response rate: the percentage of subjects with a response per CMV serostatus group. Correlation: the association between influenza and CMV antibody titers. Another outcome: influenza antibody titers were presented in context of CMV in an outcome that was not of interest for this review. Model: the effect of CMV seropositivity on influenza antibody titers was modeled and could not be extracted for this review

^1^Data were extracted from a graph reporting the average fold change on a 10log scale (mean and SEM) (outcome a)

**Fig. 4:**
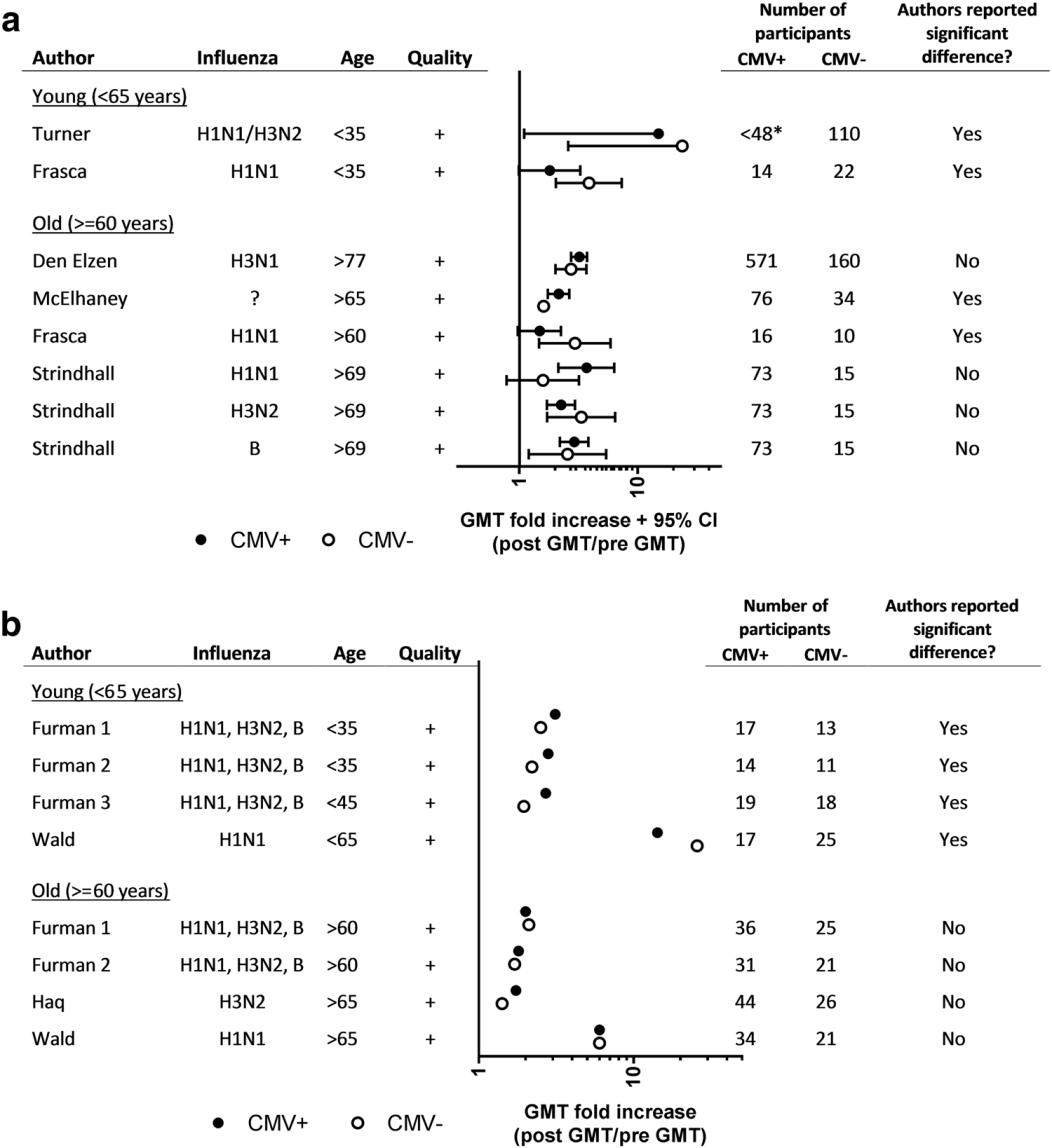
Influenza-specific geometric mean titer pre-/post-vaccination ratio (GMR) in CMV-seropositive versus CMV-seronegative participants. Studies are sorted by age of the study population (< 65 and > 60). Influenza strain, study quality and number of CMV-seropositive and CMV-seronegative participants are shown. For each outcome, it is shown whether the authors reported a significant difference between the CMV-seropositive and CMV-seronegative groups. The GMR is shown per record for CMV-seropositive (black dot) and CMV-seronegative (white dot) participants, including 95% CI error bars (**a**) or without 95% CI (**b**). *Data for Turnet et al. were not reported for CMV seropositivity (*n* = 48), but for different CMV-seropositive groups based on height of anti-CMV IgG level. Here, CMV-seropositive high individuals are shown (subgroup of *n* = 48)

^2^Data were not reported for CMV seropositivity, but for different CMV-seropositive groups based on CMV antibodies. CMV-seropositive high individuals were taken for this review and, therefore, may overestimate the effect of CMV seropositivity in Fig. 4 (outcome a)

^3^It was unclear in which Brisbane strain (H1N1 or H3N2) an effect was reported and, therefore, we could not categorize the result per influenza strain (Fig. 4) (outcome a)

^4^Data were not reported, but only stated in text that there was no effect for CMV seropositivity on the response rate (outcome b)

^5^Fold increase (of 10 log) of influenza antibodies to vaccination, and not the post-titer, was used in correlation (outcome c)

^6^For the other two influenza strains investigated in  this study, no significant correlation was found but data was not shown so not extracted for this review

^7^Data were modeled for anti-CMV IgG levels and influenza antibody titer (outcome c)

^8^Data were extracted from Table 3 of the article per vaccination strategy and calculated for total group CMV-seropositive and CMV-seronegative individuals

^9^Data were reported as the result of a model for CMV seropositivity corrected for vaccination strategy and others, and were not used for this review

^10^Median and range of influenza antibody titers were reported by this study. Of this, no outcomes of interest for this review could be extracted

^11^Data were presented as a geometric mean titer of three different influenza strains and used in this way in the analysis of this review (Fig. 4)

^12^We could not verify how the geometric mean of three influenza strains per individual was handled in the measurement of spread on group level. Confidence interval of the influenza GMR could not be extracted reliably for this review (outcome A)

^13^Confidence interval of the influenza GMR could not be extracted reliably, since error bars from the graph in the study were too small to measure (outcome A)

^14^Data were extracted from a graph reporting the average fold increase and SD (outcome a)

**Fig. 5:**
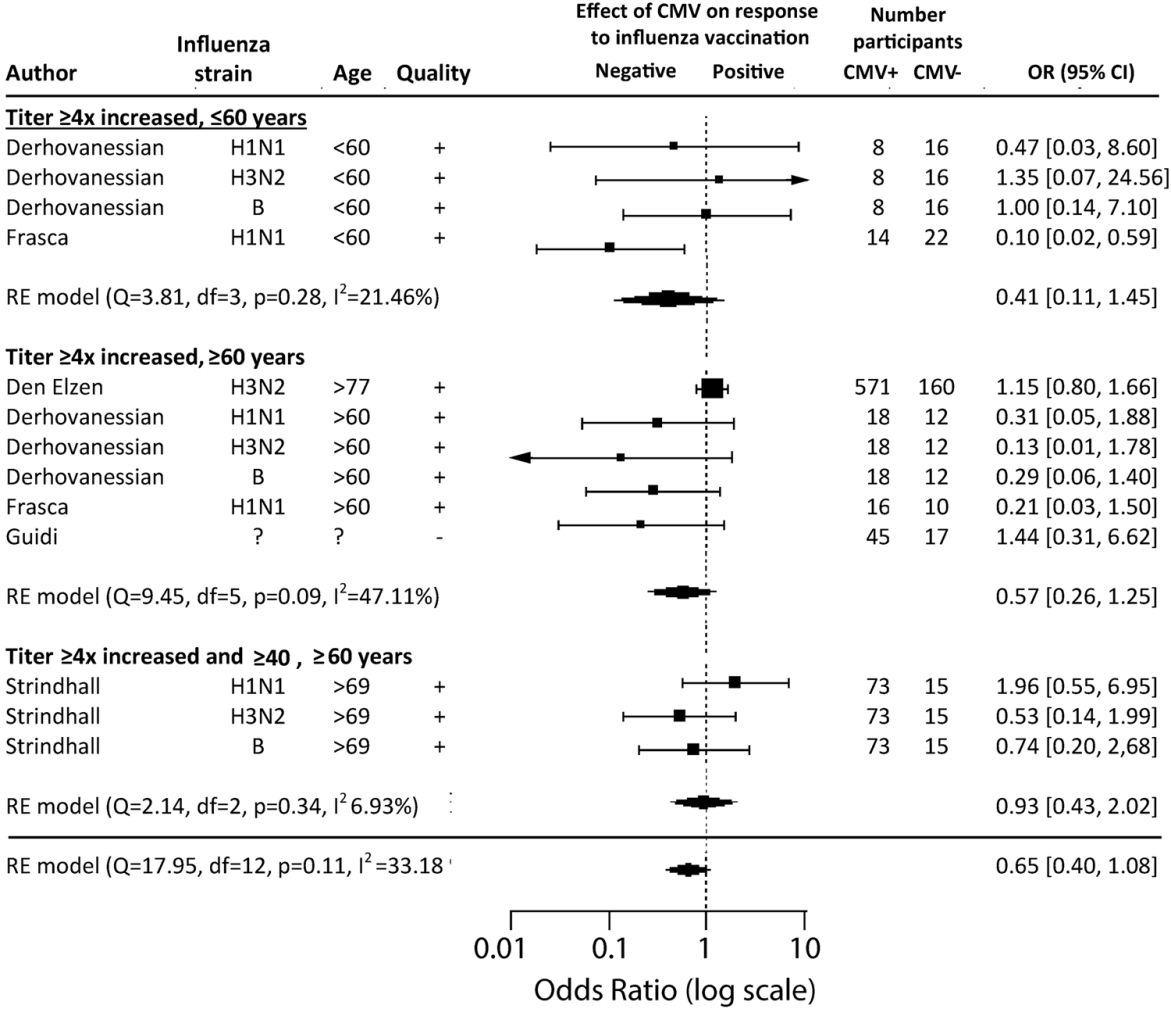
Effect of CMV serostatus on response to influenza vaccination. Results of the DerSimonian–Laird random effects model meta-analysis of five studies that included numbers of responders and non-responders to influenza vaccination. Odds ratios (diamonds) of the effect of CMV serostatus on responders to influenza and their 95% CI error bars (width of diamonds) are shown. Studies are split by age of the study population (< 60 or ≥ 60) and definition of responder that was used in the study: either ≥ four-fold increase or a four-fold increase in combination with a post-vaccination titer ≥ 40 hemagglutinating units (HAU). The influenza strain, number of study participants and overall study quality are noted for each study. *I*^2^ (the percentage of variation across studies that is due to heterogeneity rather than chance), *Q* (the weighted sum of squared differences between individual study effects and the pooled effect across studies) and *p* values (to determine whether significant heterogeneity exists) are calculated for every subgroup separately and for all studies together. Arrows indicate error bars on the odds ratio extending beyond the scale

^15^Data were extracted from a graph reporting the fold increase per individual for Fig. 5 (outcome A) and post-GMTs as stated in texts were used for Supplementary figure 5

^16^Only H1N1 data (negative effect in young and old) were shown, other measured strains were not shown because of ‘low titers’ and, therefore, not included in this review (outcome a and b)

^17^Data were analyzed with non-parametric test (Spearman correlation) on non-log-transformed antibody data (for both CMV and influenza antibodies) (outcome c)

^18^Mean CMV antibody titers were reported for responders and non-responders to influenza vaccination in this study. Of this, no outcomes of interest for this review could be extracted

^19^Data of subject with a pre-titer < 40 or ≥ 40 were pooled for this review

^20^Data were only reported as the result of a model for CMV seropositivity on combined and normalized influenza antibody titers, so no outcomes could be extracted for this review

^21^Data were extracted from Fig. 2 of the article, by extracting the GMT pre-vaccination and 21-day post-vaccination (outcome A). Although the GMT and 95% CI of day 21 post-vaccination are presented in Fig. 1 according to the article, the 95% CI showed equally distributed error bars on a linear scale, which is questionable and, therefore, no measure of spread was extracted for this review

^22^Data were not reported, but only stated in the text that there was no effect of CMV serostatus and only for the young individuals, and could therefore not be extracted for this review

^23^Data were reported in arbitrary units (based on an ELISA value divided for time elapsed since immunization) and could not be extracted for any outcome for this review

^24^Data were not reported for CMV seropositivity, but for different CMV-seropositive groups based on height of anti-CMV IgG level

^25^Data were reported as a regression model, not as correlation (outcome c)

^26^Data included the post-titer, and were extracted (outcome c)

^27^Data only included the post-GMT (Supplementary figure 5), the GMR (outcome a) could thus not be extracted for this review

### Impact of CMV on antibody titers after influenza vaccination

#### No effect of CMV-serostatus on influenza antibody titer to influenza vaccination (GMR) (outcome a)

We first examined the geometric mean titer pre-/post-vaccination ratio (GMR) to influenza vaccination for CMV-seropositive and CMV-seronegative individuals (outcome a). In 10/17 studies, the GMR per CMV serostatus could be estimated from figures [40] or could be recalculated with the reported data [26, 28, 47–51] (Supplementary figure 2). For only 5 of these 10 studies, the 95% confidence interval (CI) could be extracted [40, 47, 50–52]. These 95% CI were either estimated directly from figures or calculated based on depicted SD/SE. We refrained from calculating the ratios of the GMR of CMV-seropositive versus CMV-seronegative individuals since the measure of spread was estimated from figures and, thus, no meta-analysis was performed. Instead, we summarized the GMR with the 95% CI in an overview figure (Fig. 4a). For the remaining 5 studies, we were unable to estimate a reliable 95% CI [26, 28, 49], either due to illegible charts [49] or due to lack of clarity surrounding reported measure of spread [26, 28] (Fig. 4b).

Overall, the influenza GMR differed greatly between studies: some records suggested a higher increase in the influenza-specific antibody titer in CMV-seropositive individuals compared to CMV-seronegative individuals, while other studies suggested the opposite (Fig. 4). Even analyses restricted to young or old individuals only revealed contradicting results on the effect of CMV serostatus. Likewise, even restricting the analysis to studies of good quality revealed no overall effect of CMV seropositivity on the influenza-specific GMR after vaccination. In conclusion, the primary outcome of our systematic analysis revealed neither evidence for a negative nor for a positive effect of CMV seropositivity on the influenza vaccine response in young or old individuals.

#### Meta-analysis: no significant differences in response to influenza vaccination between CMV-seropositive and CMV-seronegative individuals (outcome b)

Next, we investigated by odds ratio (OR) analysis whether there is any evidence for a positive (OR > 1) or negative (OR < 1) association between CMV seropositivity (exposure) and response to influenza vaccination (outcome b). From five studies, a clear definition of responders and non-responders could be extracted [40, 51–54] for different influenza strains and age groups, leading to a total of 13 OR records (Supplementary figure 3). Most studies defined a response to influenza vaccination as a ≥ 4-fold increase in antibody titer after vaccination; one study (Strindhall et al., 2016) used the stricter definition of a ≥ 4-fold increase in antibody titer and a post-titer of ≥ 40 HAU. Meta-analysis of all 13 records revealed a pooled OR of 0.65 (95% CI 0.40–1.08; *I*^2^ = 33%; *p* = 0.11). Although this OR indicates a trend that CMV-seropositive participants respond less often to influenza vaccination than CMV-seronegative individuals, this is not statistically significant (Fig. 5).

Stratified meta-analyses for the separate young and old groups and for the data in Strindhall et al., which used the stricter definition of response, also did not reveal any significant effects of CMV serostatus on the influenza antibody response. A sensitivity analysis was done to assess the role of the quality of the studies on the pooled OR; meta-analysis restricted to high quality studies revealed an OR of 0.60 (95% CI 0.35–1.03), which did not markedly differ from the pooled OR of all study records. In conclusion, the average OR of 0.65 (95% CI 0.40–1.08) suggests a (non-significant) trend that CMV-seropositive participants respond less often to influenza vaccination than CMV-seronegative individuals.

#### Funnel plot analysis suggests a publication bias in meta-analysis

**Fig. 6:**
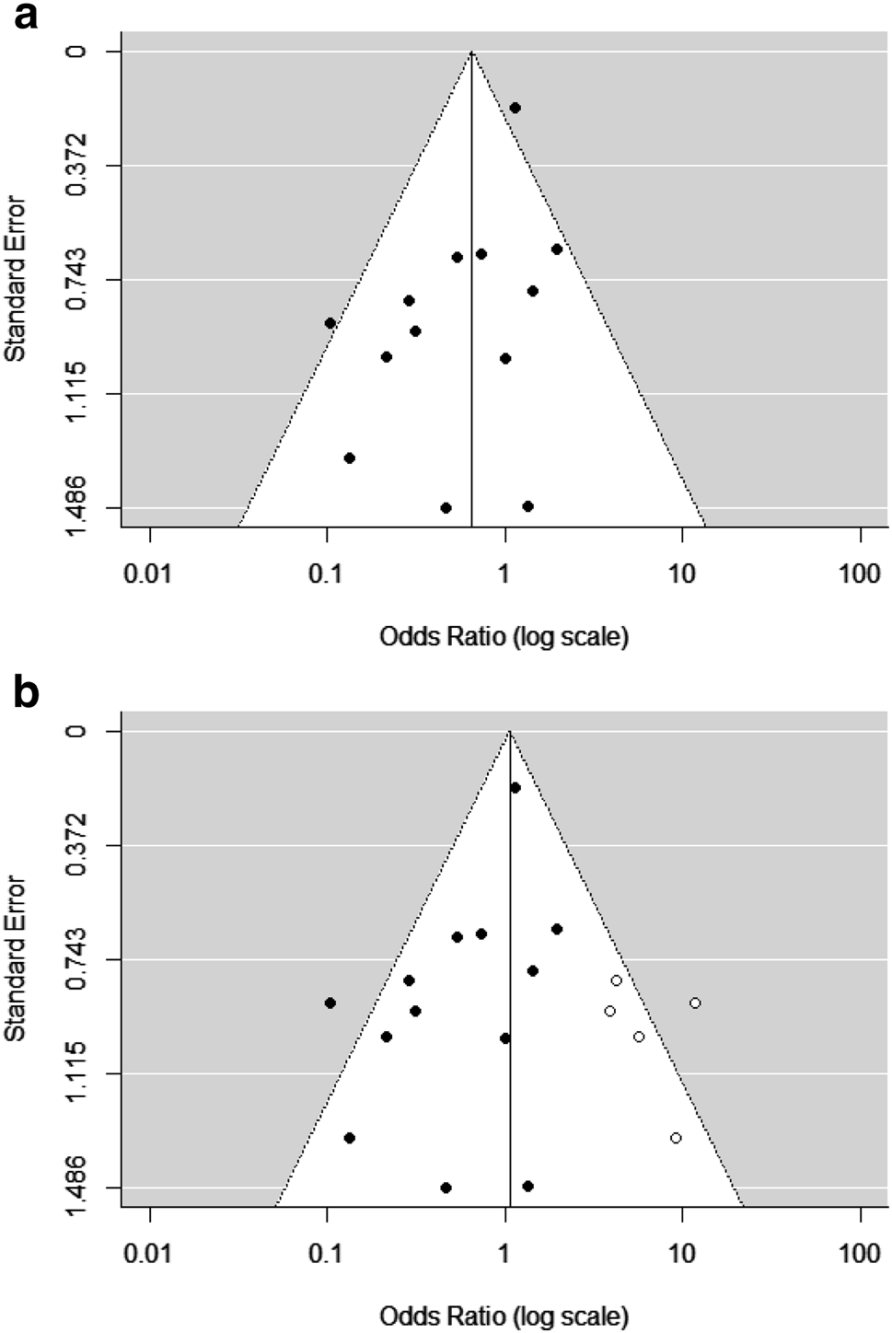
Analysis of publication bias among studies included in the meta-analysis investigating the effect of CMV serostatus on response to influenza vaccination. **a** The funnel plot shows the standard error of each study on the vertical axis (precision) and the effect size of each study (odds ratio) on the horizontal axis to assess possible asymmetry indicating publication bias. Overall pooled OR is 0.65, as indicated by the vertical line. **b** With help of the trim and fill method 5 possible unpublished studies were identified, shown as white dots. Including these hypothetical studies, the pooled OR shifts towards 1

Because a potential positive effect of CMV seropositivity on influenza vaccine responses was only recently considered, we assessed whether there was any evidence for a publication bias in the studies included in our meta-analysis by performing a funnel plot analysis [37]. Such an analysis is based on two assumptions. It assumes (1) that the OR of studies with a large study population is close to the true average OR, since they have the highest precision, while (2) the OR of studies with low precision (smaller study populations) should, based on chance, be spread evenly on both sides of the average OR. If this is not the case, there is a sign of bias in studies reaching publication. Funnel plot analysis of the studies in our meta-analysis revealed that the low precision studies reported significantly more often a negative than a positive effect of CMV (*p* = 0.019, Fig. 6a), indicating a publication bias. Importantly, influenza vaccine responses were initially investigated based on the assumption that CMV enhances immunosenescence and a positive effect of CMV on influenza antibody responses was only recently considered [28]. As a result, positive associations between CMV and the influenza vaccine response may have remained unpublished.

Based on the same two assumptions, the trim and fill method [38] was performed which removes the smaller studies causing the asymmetry, calculates the ‘true center’ of the studies and next replaces the omitted studies and missing ‘counterparts’ around the centre (filling). The trim and fill analysis estimated that five studies that revealed a positive effect of CMV-infection on the influenza vaccination response have not been published in the literature (Fig. 6b). Of note, in addition to the studies in our analysis, two more studies exist, of which the data were not published, but in which it was stated that no difference was observed [26, 47]. Interestingly, when the five hypothetical ‘missing studies’ were included in our analysis, the OR shifted up from 0.65 to 1.0, suggesting that in fact there is no effect of CMV serostatus on the response to influenza vaccination. Together, these analyses suggest that publication bias underlies the trend to a negative effect of CMV seropositivity on the response to influenza vaccination reported in the literature (Fig. 6).

#### Correlation and regression models of CMV antibody levels and influenza antibody titers suggest a negative effect of CMV-infection (outcome c)

The potential influence of latent CMV-infection on the antibody response to influenza vaccination was also investigated by extracting the association between the CMV antibody level and the influenza antibody titer. Five studies reported this outcome variable, either as a correlation between the CMV antibody level and the influenza antibody titer post-vaccination or using a regression model, in which additional factors than only CMV antibody level, such as age, were taken into account [25, 42, 47, 55, 56]. The five studies together reported 10 individual records (Supplementary figure 4) and included younger and older adults (18–97 years) and different influenza strains (Table 4).

**Table 4:** Associations reported in studies between CMV antibody titers and influenza antibody titers

Author	Quality	Type	Age	Adjusted	Association	Coefficient	*p* value
Correlation							
Turner et al. (2014)*	+	Pearson	Young (< 35)	–	CMV antibody titer and fold increase H1N1 or H3N2 titer	*r* = − 0.16	< 0.05
Trzonkowski et al. (2003)	+	Spearman	Young (< 65)	–	CMV antibody titer and H1N1 post-titer	*r* = − 0.77	0.001
		Spearman	Young (< 65)	–	CMV antibody titer and H3N2 post-titer	*r* = − 0.41	0.07
		Spearman	Young (< 65)	–	CMV antibody titer and B post-titer	*r* = − 0.39	0.08
		Spearman	Old (> 65)	–	CMV antibody titer and H1N1 post-titer	*r* = − 0.74	< 0.001
		Spearman	Old (> 65)	–	CMV antibody titer and H3N2 post-titer	*r* = − 0.41	0.03
		Spearman	Old (> 65)	–	CMV antibody titer and B post-titer	*r* = − 0.72	< 0.001
Moro-Garcia et al. (2011)	−	Spearman	Old (> 69)	–	CMV antibody titer and influenza post-vaccination titer divided by time elapsed since vaccination (strain unknown)	*r* = − 0.303	0.002
Regression models							
Turnet et al. (2014)*	+	ANCOVA model	Young (< 35)	Sex, pre-titer and pre-vaccine exercise	CMV antibody titer and fold increase H1N1 or H3N2 titer	*η*^2^ = 0.025	< 0.05
Arias et al. (2013)	−	Regression model	Old (> 69)	Age and CD8+ CD28null counts	CMV antibody titer and influenza post-vaccination titer divided by time elapsed since vaccination (strain unknown)	− 0.011	< 0.001

*Same data in Turner et al. were used for the correlation and the regression model

Unfortunately, different outcomes for associations were reported (Table 3) and raw data could not be extracted from studies to standardize the outcomes to a comparable outcome. Instead, reported associations were tabulated (Table 4). Of note, 6 out of 8 correlation outcomes came from a single study and were based on post-vaccination titers instead of the fold increase, which can be influenced to a large extent by pre-vaccination titers. For one correlation outcome, influenza antibody data were divided for time elapsed since immunization, which is not a generally accepted method. Also, both CMV-seropositive and CMV-seronegative individuals were included in all associations. For relations between anti-CMV antibody levels and influenza antibody levels, only CMV-seropositive individuals should be included in our opinion, to focus on the height of CMV antibody level as a surrogate marker of reactivation.

All records reported a negative association between the CMV antibody level and the influenza antibody titer, and 8 out of 10 were reported to be significant. Reported correlation coefficients (8 outcomes) were on average *r* = − 0.49 and varied from *r* = − 0.16 to *r* = − 0.77, showing mainly low to moderate negative correlations [57]. In addition, two models [42, 47] showed a significant negative association between CMV antibody levels and the influenza antibody titer to influenza vaccination. Overall, these correlation results indicate a small but significant negative association between CMV antibody levels and influenza antibody titers after vaccination.

## Discussion

This is the first systematic review investigating the association between latent CMV infection and the immune response to influenza vaccination. Almost two decades ago, CMV was associated with “immunosenescence” [58]. Since then, multiple studies on CMV-induced immunosenescence have been performed. The idea that CMV decreases the ability of the immune system to respond to other pathogens or vaccination [59, 60] is mainly based on studies investigating the influenza vaccine response [61]. Indeed, various studies reported a negative association between latent CMV infection and influenza vaccine responses, while other studies lacked to find an effect of CMV or even reported a positive effect of CMV. Thus, consensus on the effect of CMV is lacking. Nevertheless, an effect of CMV on the influenza vaccine response in the elderly is generally assumed [29, 61, 62]. By systematically reviewing and integrating the available studies, we here show that there is no unequivocal evidence for an impact of CMV on the influenza vaccine response.

We systematically selected studies on CMV and influenza vaccine responses and extracted three standardized influenza antibody outcome variables. The geometric mean titer ratio (GMR) pre/post-influenza vaccination with (Fig. 4a) and without (Fig. 4b) 95% CI revealed no difference between CMV-seropositive and CMV-seronegative individuals (outcome a). Of note, also when only the post-vaccination geometric mean titer (post-GMT) was summarized, no overall trend for an effect of CMV serostatus was observed (Supplementary figure 5). We primarily assessed the GMR and not the post-GMT since the participants in the studies were not all influenza seronegative before vaccination. Pre-existing immunity is usually present in the case of seasonal influenza vaccination. Thus, post-vaccination titers as outcome will overestimate the vaccine antibody response and are, therefore, less meaningful. Linear regression analysis, as performed in some studies [42, 63], is the best method to correct for pre-vaccination titers [46], but this could not be analyzed on the basis of the extracted data of the studies included for this review. Thus, with the GMR, the best outcome available, no effect of CMV seropositivity on the influenza vaccine response is observed.

The meta-analysis of response rate to influenza vaccination (outcome b) (Fig. 5) revealed a small (albeit non-significant) trend that CMV-seropositive participants respond less often to influenza vaccination than CMV-seronegative individuals. Funnel plot analysis suggested that publication bias most likely underlies this trend in the literature (Fig. 6).

Unfortunately, it was not possible to extract a standardized outcome for an association between CMV antibody level and the influenza antibody response to vaccination (outcome c), since the methods of the studies varied and no raw data were available. Overall, the reported correlation results (Table 4) of the studies indicated a small negative association between CMV antibody titers levels and influenza antibody levels after vaccination, suggesting that individuals who experienced multiple CMV reactivations during life may have impaired influenza vaccine responses. The tabulated correlations, however, should be interpreted with caution. CMV antibody levels increase with age and are thought to reflect experienced CMV reactivation or reinfection [7]. Therefore, high anti-CMV antibody levels may be related to enhanced CMV-induced immunosenescence and impaired influenza vaccine responses [42]. However, we noticed that in most studies CMV-seronegative individuals were included in the correlation of CMV antibody titers and influenza antibody titers, which may affect the correlation coefficient or the significance of the correlation. Of importance, in only one study CMV antibody levels were correlated with the fold increase in influenza antibody titers [47]; in all other studies, it was correlated with the post-vaccination titer, thereby overestimating the vaccine response. Together, this questions the importance of the reported weak correlations between CMV antibody levels and influenza antibody titers to vaccination.

To illustrate the controversy in the literature, we also summarized the reported conclusions of various studies on the influenza antibody response (Fig. 3). Two previous reviews directly combined the various results in literature on the effect of latent CMV-infection on the antibody response to influenza vaccination [4, 29]. Frasca et al. (2015) and Merani et al. (2017) refer to some of the studies included in our systematic review and describe the effect of CMV on influenza antibody vaccine responses as controversial or ambiguous. Despite this, both reviews come to the conclusion that CMV does affect the immune response to influenza vaccination [4, 29]. In addition, Merani et al. discuss possible methods to reduce the impact of immunosenescence on influenza vaccine responses by anti-CMV strategies [29]. The controversy in the literature and the difficulty to compare different influenza antibody outcomes in different studies highlight that a systematic approach is necessary.

The strength of our review lies in its systematic approach. This allowed us to synthesize all the available evidence (until 27th June 2017) on this particular question and to eliminate the effect of potential publication bias. Instead of merely summarizing the conclusions in literature, we extracted the published data in three standardized outcome variables of influenza antibody response, separated per age group. Furthermore, whenever possible, we assessed the data of each study per influenza strain. By this, we included multiple records per study and not only the record on which the authors’ conclusion was based. To the best of our knowledge, only two new articles came out that investigated the effect of latent CMV infection on the influenza antibody response since the systematic search of this review (27th June 2017). Merani et al. (sept 2017) concluded that there is no difference in influenza GMR between CMV-seronegative and CMV-seropositive individuals, while CMV-seropositive individuals do show an impaired cellular granzyme B response to influenza virus challenge [64]. We published in Van den Berg et al. (January 2018) that there is no negative effect of CMV infection on the antibody response to a novel influenza vaccine strain in adults [65]. Both studies will not change the conclusion of this systematic review that there is no unequivocal evidence for an effect of CMV infection on the antibody response to influenza vaccination.

In this review, the direct association between CMV infection and the influenza antibody vaccine response is investigated. Several mechanisms of a potential negative effect have been postulated, based on the known effects of CMV infection on the immune system and the subsequent potential impact on the influenza vaccine response [47, 58]. CMV infection leads to increased pro-inflammatory cytokine levels, which in turn are associated with decreased influenza vaccine responses [29]. Likewise, CMV infection leads to increased differentiation of T cells, which has been associated with poor influenza vaccination responses [25, 53]. It has also been reported that CMV infection is associated with decreased switched B cell percentages before influenza vaccination, and subsequently lower influenza vaccine responses [32]. In contrast, De Bourcy et al. (2017) suggested a potential mechanism for the positive effect of CMV on the antibody influenza vaccine response reported in Furman et al.: based on B-cell repertoire analyses, CMV infection is associated with more activated B cells after influenza vaccination.

This systematic review also has some limitations. Ideally, a systematic review is based on randomized controlled trials (RCT), but due to obvious ethical and practical reasons, no RCTs have been conducted to study the relation between CMV infection and influenza vaccine responses. Consequently, only observational studies were included. We assumed that the reported sizes of the study populations were correct for the duration of the studies, even if no statement was made on the number of participants that were lost to follow-up. Furthermore, this systematic review was limited by the number of studies that was found to be eligible for inclusion, which led to a meta-analysis of only 5 studies leading to 13 records. Another limitation is the incomplete correcting for confounders. We only adjusted for pre-vaccination antibody levels by investigating the GMR and partially adjusted for age by separating the results for young and old adults. However, age is associated with different influenza antibody responses, not only because of immunosenescence, but also due to immunologic imprinting and different influenza exposure during lifetime. Thus, merely assigning individuals to a young and old age group might not be sufficient to adjust for age as a confounder. Research on the effect of CMV on the influenza antibody vaccine response is further complicated by different study populations, and different influenza strains, as summarized previously [29]. There is no biological basis for a differential effect of CMV on different influenza strains, but influenza vaccine responses vary a lot per season and subtype [66]. Unfortunately, data from Reed et al. could not be incorporated in this review. They reported a negative association between CMV seropositivity and influenza antibody vaccine response in a study including different seasons and influenza strains. Their data could, however, not be extracted for analysis for one of the outcomes of this systematic review, since they were only reported as a result of a multi-factor model.

Universality in reported influenza antibody data in the CMV-immunosenescence field is necessary to reveal the potential effect of CMV on the antibody influenza vaccine response. We recommend further studies investigating the effect of CMV-infection on the influenza antibody vaccine response to follow the EMA guidelines [24] and as an absolute minimum, to always report the influenza pre-GMT and post-GMT (with 95% CI) and the number of participants per group. It is important to take influenza strain and season into account by measuring and reporting the titers separately per influenza strain. A response rate is also of interest, but should not be the only outcome reported. The response rate can be defined in several ways [24] and the correlate of protection of 40 is based on adults, making the use in elderly questionable [24, 67]. In addition, a regression analysis to correct for pre-existing immunity is necessary. Especially when the effect of CMV infection on the influenza antibody response is small, correcting for confounders, like age (as continuous variable), pre-existing immunity, vaccination history, medicine use or comorbidities is highly recommended.

In conclusion, we show that based on the GMR, which in our perception is the best outcome available, there is no evidence for an effect of CMV seropositivity on the influenza antibody vaccine response, and that publication bias probably explains the trend in the literature that CMV-seropositive individuals seem to respond less often to influenza vaccines than CMV-seronegative individuals. We suspect that in the past, several studies did not reach publication because they did not fit the prevailing idea that CMV induces immunosenescence. Our systematic review emphasizes that the effect of CMV infection on a clinically relevant immune function in humans, such as influenza vaccine responses, is not as black-and-white as previously suggested. Further large studies investigating the relation between CMV antibody levels and influenza vaccine responses with enough power to detect a potential small effect of CMV infection are needed, in which also confounding factors in addition to age are taken into account. Only if there is unequivocal evidence for CMV-associated impaired influenza vaccine responses, can we begin to address whether a CMV-impaired vaccine response in the elderly is merely a sign of immunosenescence, or whether CMV is causing immunosenescence.

## Electronic supplementary material

Below is the link to the electronic supplementary material.

Supplementary material 1 (PDF 521 kb)

Supplementary material 2 (PDF 99 kb)
