# Supplementary material for: Effect of latent cytomegalovirus infection on the antibody response to influenza vaccination: a systematic review and meta-analysis
Source: Med Microbiol Immunol. 2019 Apr 4;208(3):305–21. doi: 10.1007/s00430-019-00602-z (PMC6647367; doi:10.1007/s00430-019-00602-z)
Supplement: Supplementary file 1 — Supplementary material 1 (PDF 521 kb) [file 430_2019_602_MOESM1_ESM.pdf]

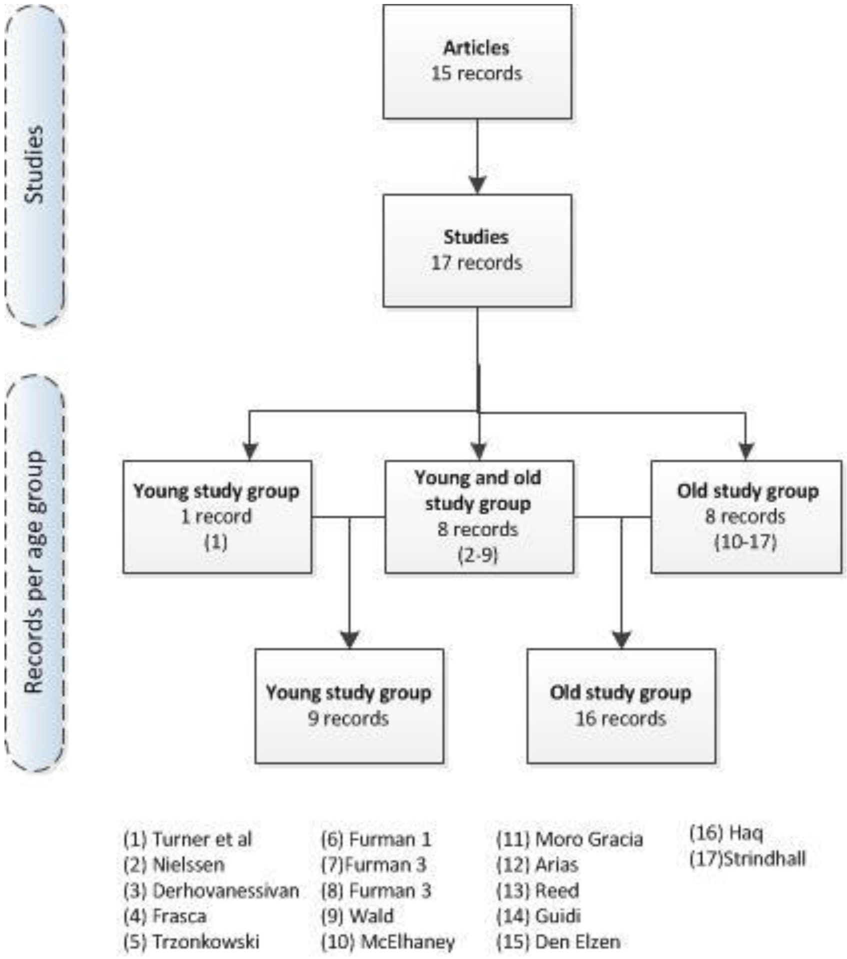

Supplementary figure 1. Flow diagram of number of records available for reported conclusions of the effect of CMV on influenza antibody response in young (<60 years of age) or old (>60 years of age) adults.

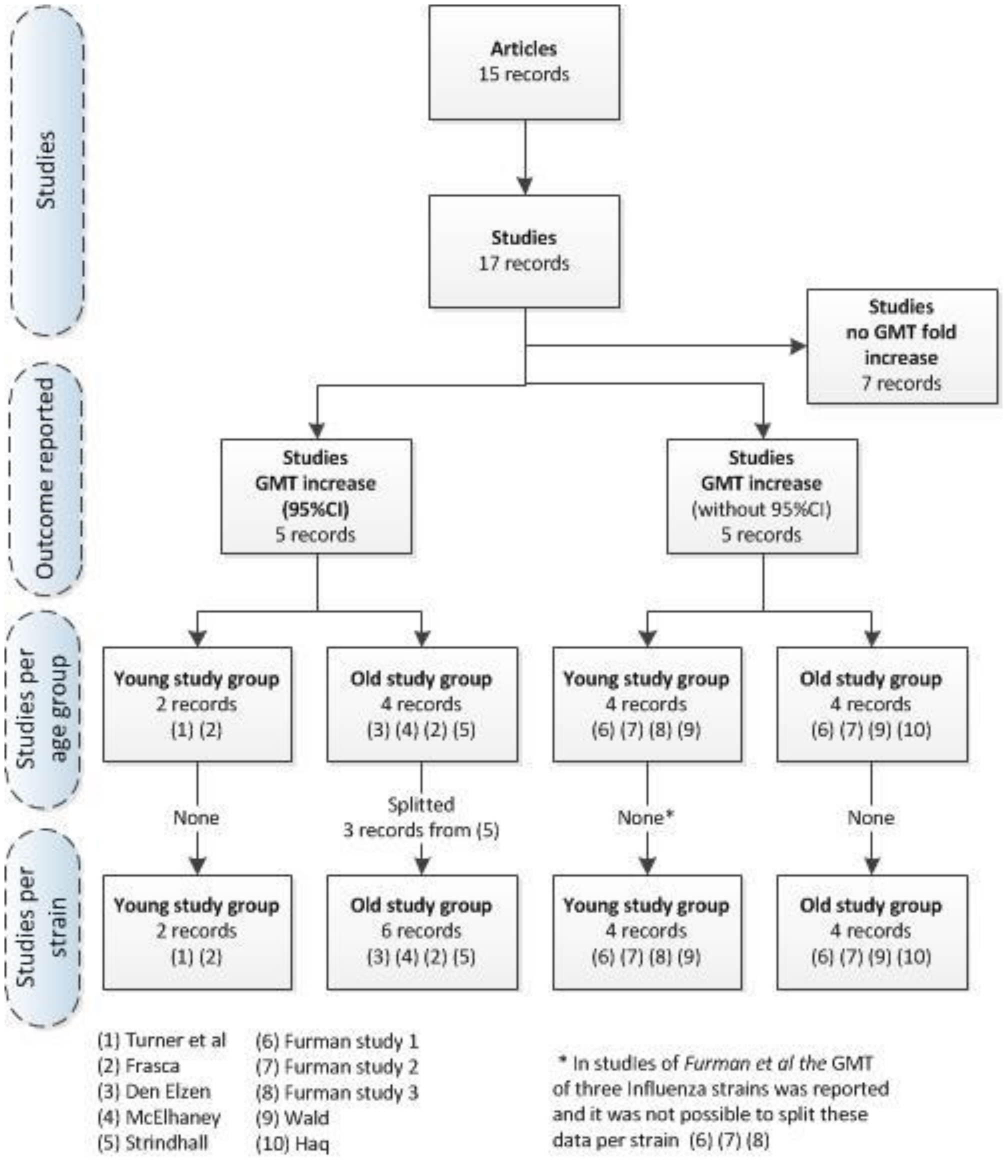

Supplementary figure 2. Flow diagram of number of records available for influenza GMT fold increase (outcome A) with and without 95% confidence interval (CI).

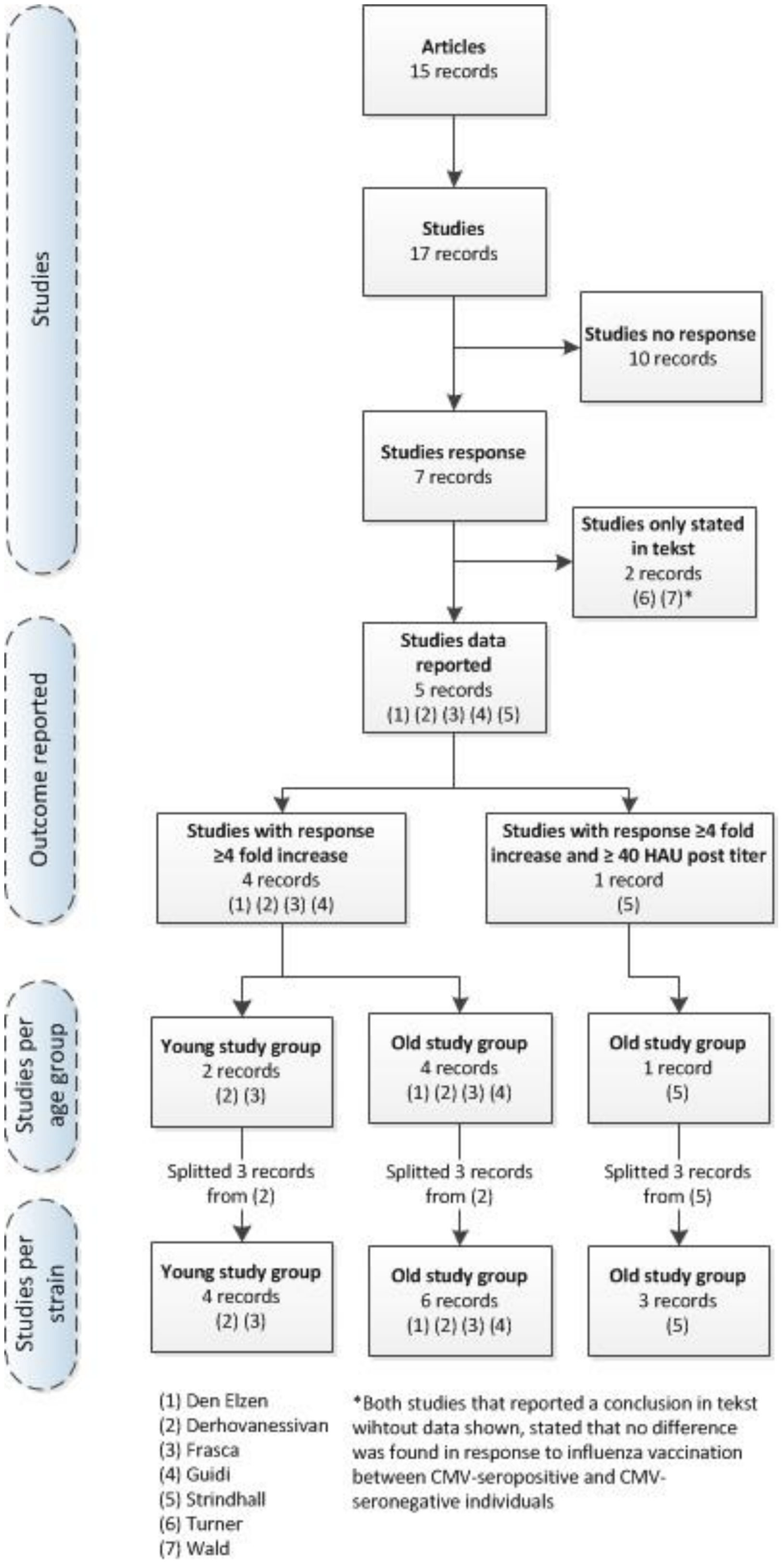

Supplementary figure 3. Flow diagram of number of records available for effect of CMV-serostatus on response to influenza vaccination (outcome B)

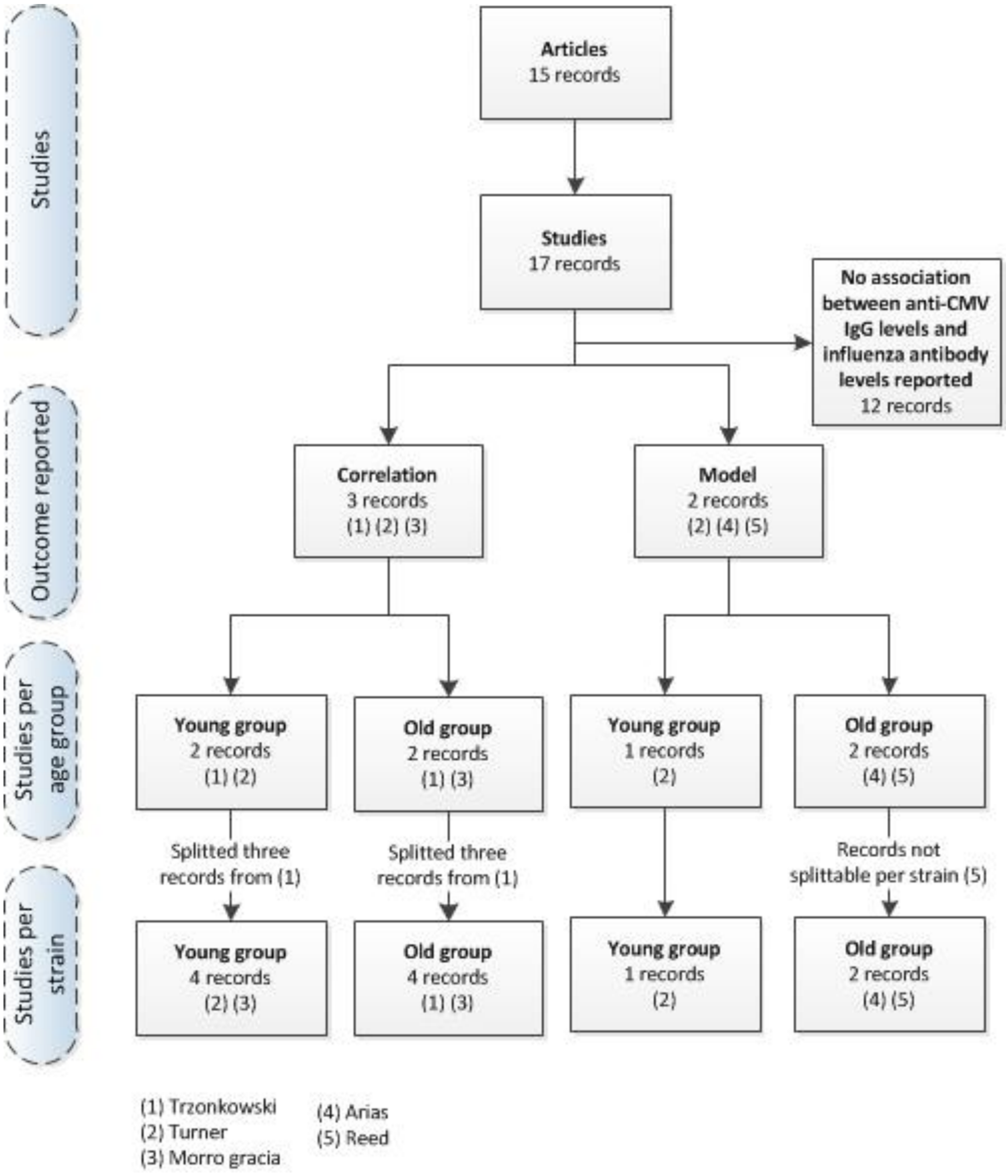

Supplementary figure 4. Flow diagram of number of records available for correlation influenza antibody titers and CMV IgG levels (outcome C)

Author	Influenza	Age	Quality	Number participants CMV+	Number participants CMV-	Authors reported significant difference
With 95% CI						
Den Elzen	H3N1	>77	+	<48*	110	No
Strindhall	H1N1	>69	+/-	73	15	No
Strindhall	H3N2	>69	+/-	73	15	No
Strindhall	B	>69	+/-	73	15	No
Without 95% CI						
Wald	H1N1	<65	-	17	25	Yes
Frasca	H1N1	<35	+	14	22	Yes
Wald	H1N1	>65	-	34	21	No
Frasca	H1N1	>60	+	16	10	Yes
Haq	H3N2	>65	+/-	44	26	No
Guidi	?	?	--	54	11	No

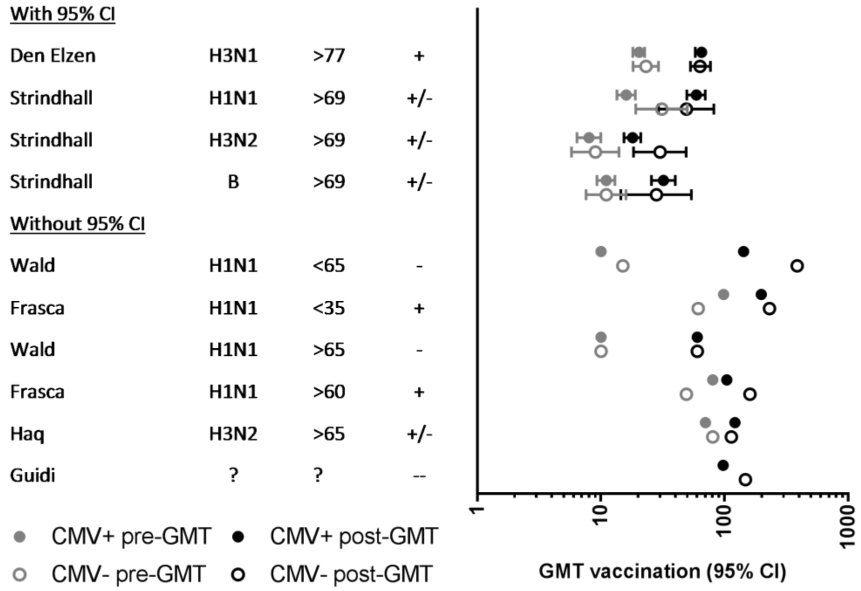

Supplementary figure 5. Pre-vaccination geometric mean titer (pre-GMT) and post-vaccination geometric mean titer (post-GMT) in CMV-seropositive versus CMV-seronegative participants. * Data for *Turnet er al* was not reported for CMV-seropositivity (n=48), but for different CMV-seropositive groups based on height of anti-CMV IgG level. Here, CMV-seropositive high individuals are shown (subgroup of n=48).
