# Supplementary material for: Effect of latent cytomegalovirus infection on the antibody response to influenza vaccination: a systematic review and meta-analysis
Source: Med Microbiol Immunol. 2019 Apr 4;208(3):305–21. doi: 10.1007/s00430-019-00602-z (PMC6647367; doi:10.1007/s00430-019-00602-z)
Supplement: Supplementary file 2 — Supplementary material 2 (PDF 99 kb) [file 430_2019_602_MOESM2_ESM.pdf]

Number	Query results	Results
#27	'cytomegalovirus infection'/exp AND 'influenza vaccine'/exp OR ('cytomegalovirus infection'/exp AND 'influenza vaccination'/exp) OR ('cytomegalovirus infection'/exp AND ('influenza virus'/exp AND 'vaccine'/exp OR ('influenza virus'/exp AND 'vaccination'/exp))) OR ('cytomegalovirus'/exp AND 'influenza vaccine'/exp) OR ('cytomegalovirus'/exp AND 'influenza vaccination'/exp) OR ('cytomegalovirus'/exp AND ('influenza virus'/exp AND 'vaccine'/exp OR ('influenza virus'/exp AND 'vaccination'/exp))) OR ('cytomegalovirus'/exp/mj AND ('immune response'/exp/mj OR 'immune status'/exp/mj)) OR ('cytomegalovirus infection'/exp/mj AND ('immune response'/exp/mj OR 'immune status'/exp/mj)) AND 'human'/de	689
#26	'cytomegalovirus infection'/exp AND 'influenza vaccine'/exp OR ('cytomegalovirus infection'/exp AND 'influenza vaccination'/exp) OR ('cytomegalovirus infection'/exp AND ('influenza virus'/exp AND 'vaccine'/exp OR ('influenza virus'/exp AND 'vaccination'/exp))) OR ('cytomegalovirus'/exp AND 'influenza vaccine'/exp) OR ('cytomegalovirus'/exp AND 'influenza vaccination'/exp) OR ('cytomegalovirus'/exp AND ('influenza virus'/exp AND 'vaccine'/exp OR ('influenza virus'/exp AND 'vaccination'/exp))) OR ('cytomegalovirus'/exp/mj AND ('immune response'/exp/mj OR 'immune status'/exp/mj)) OR ('cytomegalovirus infection'/exp/mj AND ('immune response'/exp/mj OR 'immune status'/exp/mj))	837
#25	'cytomegalovirus'/exp/mj AND ('immune response'/exp/mj OR 'immune status'/exp/mj) OR ('cytomegalovirus infection'/exp/mj AND ('immune response'/exp/mj OR 'immune status'/exp/mj))	429
#24	'cytomegalovirus infection'/exp/mj AND ('immune response'/exp/mj OR 'immune status'/exp/mj)	227
#23	'cytomegalovirus'/exp/mj AND ('immune response'/exp/mj OR 'immune status'/exp/mj)	238
#22	'immune response'/exp/mj OR 'immune status'/exp/mj	92,574
#21	'cytomegalovirus'/exp/mj	14,375
#20	'cytomegalovirus infection'/exp/mj	16,489
#19	'immune status'/exp/mj	932
#18	'immune response'/exp/mj	91,662
#17	'cytomegalovirus infection'/exp AND 'influenza vaccine'/exp OR ('cytomegalovirus infection'/exp AND 'influenza vaccination'/exp) OR ('cytomegalovirus infection'/exp AND ('influenza virus'/exp AND 'vaccine'/exp OR ('influenza virus'/exp AND 'vaccination'/exp))) OR ('cytomegalovirus'/exp AND 'influenza vaccine'/exp) OR ('cytomegalovirus'/exp AND 'influenza vaccination'/exp) OR ('cytomegalovirus'/exp AND ('influenza virus'/exp AND 'vaccine'/exp OR ('influenza virus'/exp AND 'vaccination'/exp)))	414
#16	'cytomegalovirus'/exp AND ('influenza virus'/exp AND 'vaccine'/exp OR ('influenza virus'/exp AND 'vaccination'/exp))	122
#15	'cytomegalovirus'/exp AND 'influenza vaccination'/exp	41
#14	'cytomegalovirus'/exp AND 'influenza vaccine'/exp	134
#13	'cytomegalovirus infection'/exp AND ('influenza virus'/exp AND 'vaccine'/exp OR ('influenza virus'/exp AND 'vaccination'/exp))	47
#12	'cytomegalovirus infection'/exp AND 'influenza vaccination'/exp	72
#11	'cytomegalovirus infection'/exp AND 'influenza vaccine'/exp	203
#10	'influenza virus'/exp AND 'vaccine'/exp OR('influenza virus'/exp AND 'vaccination'/exp)	13,079
#9	'influenza virus'/exp AND 'vaccination'/exp	6,925
#8	'influenza virus'/exp AND 'vaccine'/exp	11,990
#7	'vaccine'/exp	286,298
#6	'vaccination'/exp	143,378
#5	'influenza virus'/exp	54,474
#4	'influenza vaccination'/exp	13,281
#3	'influenza vaccine'/exp	30,951
#2	'cytomegalovirus'/exp	34,775
#1	'cytomegalovirus infection'/exp	34,353

Supplementary Table 1. Literature search strategy for the effect of CMV-infection on the immune response to influenza vaccination. Results represent the number of articles. Search was performed on 27 june 2017.

Data extraction form per study

Characteristics of studies (Summarized in Table 1)

Primary study design	Design for research question	Original paper URL	Conclusion/ summary	Population (sample size and age)	Exclusion criteria	Setting (country etc)	Timeline

Influenza vaccine outcomes (Summarized in Table 2)

Increase GMT (outcome 1)	Response (outcome 2)	Correlation (outcome 3)	Antibodies (another way)	Model (corrected)	Cellular

Conclusion articles (Summarized in Figure 3, and Supplementary table 3)

Influenza virus strain(s) antibodies measured	Reported effect of CMV Young study group	Reported effect of CMV Old study group	Statistical method

Risk of bias and study quality of studies included for systematic assessment (summarized in Table 3)

Study controls for	Statistical method appropriate	Selection (max 4*)	Comparability (max 2*)	Outcome (max 3*)	Overall score (+/-)

Influenza vaccination (summarized in Supplementary table 4)

Type vaccine	year and pandamic or seasonal	Dose	influenza virus strain(s)	Booster

Time and type of measurement of influenza and CMV antibodies (summarized in Supplementary table 5)

Influenza antibody measurement			CMV antibody measurement		
Time after vaccination	Assay of measurement	Vaccination history	Assay of measurement	Cut-off CMV-seropositive	Borderline handling

Supplementary Table 2. Data extraction form per study.

Study	Influenza virus strain(s) antibodies measured	Reported effect of CMV<br><br>Young	Reported effect of CMV<br><br>Old
**Turner et al,** 2014	A/H3N2<br>A/H1N1<br>A/H1N1<br>B Vic	**Negative**<br>Brisbane H1N1 or H3N2*<br>**No effect**<br>Brisbane H1N1 or H3N2*<br>B Vic	-
**Den Elzen et al**, 2011	A/H3N2	-	**No effect**<br>A/H3N2
**Derhovanessian et al**, 2012	A/H1N1<br>A/H3N2<br>B Vic	**No effect**<br>A/H1N1<br>A/H3N2<br>B Vic	**Negative**<br>A/H3N2<br>**No effect**<br>A/H1N1<br>B Vic
**Nielsen et al**, 2015	A/H1N1	**No effect**<br>A/H1N1 (age range 21-77)	
**Furman et al**, 2015 – study 1 **	A/H1N1<br>A/H3N2<br>B Vic	**Positive**<br>GM of the strains A/H1N1, A/H3N2, B Vic	**No effect**<br>GM of the strains A/H1N1, A/H3N2, B Vic
**Furman et al**, 2015 – study 2 **	A/H1N1<br>A/H3N2<br>B Vic	**Positive**<br>GM of the strains A/H1N1, A/H3N2, B Vic	**No effect**<br>GM of the strains A/H1N1, A/H3N2, B Vic
**Furman et al**, 2015 – study 3 **	A/H1N1<br>A/H3N2<br>B Vic	**Positive**<br>GM of the strains A/H1N1, A/H3N2, B Vic	**No effect**<br>GM of the strains A/H1N1, A/H3N2, B Vic
**Haq et al**, 2016	A/H3N2	-	**No effect**<br>A/H3N2
**McElhaney et al**, 2015	Unkown which strain was measured	-	**Positive**<br>Unkown which strain
**Frasca et al**, 2015	A/H1N1<br>A/H3N2<br>B Vic	**Negative**<br>A/H1N1<br>Others were not shown 'because low titers'	**Negative**<br>A/H1N1<br>Others were not shown 'because low titers'
**Trzonkowski et al**, 2003	A/H1N1<br>A/H3N2<br>B Yam	**Negative**<br>H1N1<br>**No effect**<br>A/H3N2<br>B Yam<br>(not significant: P=0.07 and P=0.08)	**Negative**<br>A/H1N1<br>A/H3N2<br>B Yam2<br>1
**Strindhall et al**, 2015	A/H1N1<br>A/H3N2<br>B Vic	-	**No effect**<br>A/H1N1<br>A/H3N2<br>B Vic
**Reed et al**, 2016	A/H1N1<br>A/H3N2<br>B<br>(For each year performed)	-	**Negative**<br>Averaged strains per year. On persistence and on peak in combination with other factors.
**Wald et al**, 2013	A/H1N1pdm	**Positive**<br>A/H1N1pdm	**No effect**<br>A/H1N1pdm

**Arias et al**, 201	Unkown which strain was measured	-	**Negative**<br>Unkown
**Moro-Garcia et al**, 2011	Unkown which strain was measured	-	**Negative**<br>Unkown
**Guidi et al**, 2014	A/H1N1 or A/H1N1pdm	-	**No effect**<br>A/H1N1 or A/H1N1pdm

* Unkown for which influenza A/Brisbane strain a negative effect respectively no effect was found in this study (H1N1 or H3N2)

- not investigated in this study for this age group.

** Studies from Furman et al (1) reported a geometric mean of three influenza strains, no data of individual strains was reported.

Supplementary Table 3. Conclusion reported by articles.
